# Targeting EpCAM by a Bispecific Trifunctional Antibody Exerts Profound Cytotoxic Efficacy in Germ Cell Tumor Cell Lines

**DOI:** 10.3390/cancers12051279

**Published:** 2020-05-19

**Authors:** Stefan Schönberger, Daniela Kraft, Daniel Nettersheim, Hubert Schorle, Anna Casati, Rogerio B. Craveiro, Mahsa Mir Mohseni, Gabriele Calaminus, Dagmar Dilloo

**Affiliations:** 1 Department of Pediatric Hematology and Oncology, University Hospital Bonn, University of Bonn, 53127 Bonn, Germany; casati.anna77@gmail.com (A.C.); mahsa.mir_mohseni@ukbonn.de (M.M.M.); gabriele.calaminus@ukbonn.de (G.C.); dagmar.dilloo@ukbonn.de (D.D.); 2 Department of Pediatric Hematology and Oncology, University Hospital Essen, University of Essen, 45147 Essen, Germany; 3 Clinipace, Clinical Research Organisation, European Headquarters Eschborn, 65760 Eschborn, Germany; dkraft@clinipace.com; 4 Department of Urology, Urological Research Lab, Translational UroOncology, University Hospital Düsseldorf, 40225 Düsseldorf, Germany; daniel.nettersheim@med.uni-duesseldorf.de; 5 Institute of Pathology, Department of Developmental Pathology, University Hospital Bonn, University of Bonn, 53127 Bonn, Germany; hubert.schorle@ukbonn.de; 6 Department of Orthodontics, University Hospital of RWTH Aachen, University of Aachen, 52074 Aachen, Germany; rcraveiro@ukaachen.de

**Keywords:** EpCAM, CD3, bispecific antibody, immunotherapy, Catumaxomab, germ cell tumors, embryonal carcinoma, seminoma, choriocarcinoma

## Abstract

Outcome in high-risk patients with refractory or relapsed germ cell tumours (GCT) remains poor. Novel strategies enhancing therapeutic efficacy whilst limiting therapeutic burden are warranted, yet immunotherapy approaches geared towards activating endogenous antitumor responses have not been successful thus far. Redirection of cytotoxic effector cells by bispecific antibodies represents a promising approach in this setting. We demonstrate that the Epithelial Cell Adhesion Molecule (EpCAM) is broadly expressed in GCT cell lines of different histologic origin including seminoma, choriocarcinoma (CHC), and embryonal carcinoma (EC). In these GCT lines of variable EpCAM surface expression, targeting T cells by the prototypic bispecific EpCAM/CD3-antibody (bAb) Catumaxomab together with natural killer (NK) cell engagement via the Fc domain promotes profound cytotoxicity across a broad range of antibody dilutions. In contrast, tumor cell lysis mediated by either immune cell subset alone is influenced by surface density of the target antigen. In the CHC line JAR, NK cell-dependent cytotoxicity dominates, which may be attributed to differential surface expression of immunomodulatory proteins such as MHC-I, CD24, and Fas receptors on CHC and EC. In view of redirecting T cell therapy mediated by bispecific antibodies, such differences in GCT immunophenotype potentially favoring immune escape are worth further investigation.

## 1. Introduction

Malignant Germ cell tumors (GCT) constitute a heterogeneous group of tumors comprising undifferentiated seminoma as well as embryonal carcinoma (EC), choriocarcinoma (CHC), yolk sac tumor (YST), or teratoma components [1]. GCT are thought to be derived from a common embryologic origin, with potential differences in the maturation stage of the cells transformed. In young adult males, testicular GCT presents the most frequent malignancy, while in children, GCT are rare and also localize to extragonadal sites [2,3]. Ever since cisplatin has become an integral part of systemic therapy, prognosis has consistently improved and is currently excellent for seminomatous and favorable for non-seminomatous GCT. In contrast, outcome in high-risk patients with cisplatin-refractory or recurrent disease remains poor [4,5,6], warranting novel therapeutic approaches. In addition, platinum-based chemotherapy is associated with potentially severe toxicity and long-term sequelae affecting outcome with considerable consequences [7,8,9]. Thus, tailored therapy increasing therapeutic efficacy in high-risk patients whilst limiting therapeutic burden is urgently needed.

In cancer therapy, improved understanding of interactions between tumor and immune cells has paved the way for establishing effective immunotherapeutic approaches. While in the past GCT have long been considered to mimic testicular immune privilege, consolidating evidence has revealed infiltration of activated cytotoxic and memory T cells as well as macrophages predominately in seminoma with helper T cells and natural killer (NK) cells being rarely present [10,11,12,13]. Although preliminary immunotherapy trials with immune checkpoint inhibitors have been initiated with the aim to overcome immune escape, efficacy has been limited as yet [14,15]. Of note, beyond these efforts, data from either in vitro or in vivo studies exploring the susceptibility of GCT to immune cell-mediated cytotoxicity are sparse.

Amongst bispecific antibodies (bAb), constructs that comprise dual binding sites for both CD3 and a suitable target antigen on tumor cells are attracting increasing attention as strategies of antineoplastic immunotherapy. They redirect T cells to tumor cells promoting cytotoxic synapse formation and subsequent tumor cell lysis. In addition, via their Fc-domain, bAb enlist accessory cells such as natural killer (NK) cells and macrophages into the immune response, complementing T cell-dependent tumor destruction [16].

In GCT, the transmembrane epithelial adhesion molecule (EpCAM; CD326) constitutes a promising target for such an approach as it is broadly present on the surface of GCT of different origins irrespective of age, sex, site, and clinical tumor stage [17]. EpCAM expression increases with the degree of teratoma immaturity, is detectable on EC, and is particularly high in CHC and YST [17]. After intramembrane proteolysis, the intracellular EpCAM domain EpiCD associates with elements of the WNT pathway enhancing gene transcription, cell proliferation, and tumor formation and progression [18]. Moreover, we and others have previously documented upregulation of WNT signaling in GCT of distinct histologies corresponding to variable levels of immune cell infiltration observed in this group [19,20]. It is current knowledge that, in cancer, activated WNT signaling and ß-Catenin accumulation is associated with T cell exclusion from the tumor microenvironment and down-modulation of T cell cross-priming [20,21]. In this setting, redirection and activation of T cells by bAb recognizing antigens expressed on the tumor cell surface bypasses limitations of MHC-restricted tumor-antigen detection and promotes cytotoxic effector cell attack through generation of a pro-inflammatory milieu.

Here, we demonstrate that, in GCT cell lines of different histology, the EpCAM-directed prototypic bAb Catumaxomab facilitates recruitment and activation of accessory cells in addition to redirected T cells and promotes highly efficacious antineoplastic toxicity. We also delineate characteristics in GCT phenotype potentially favoring immune escape that are worth further investigation.

## 2. Results

### 2.1. EpCAM Is Expressed in Seminomatous as Well as Non-Seminomatous GCT Cell Lines

**Figure 1:**
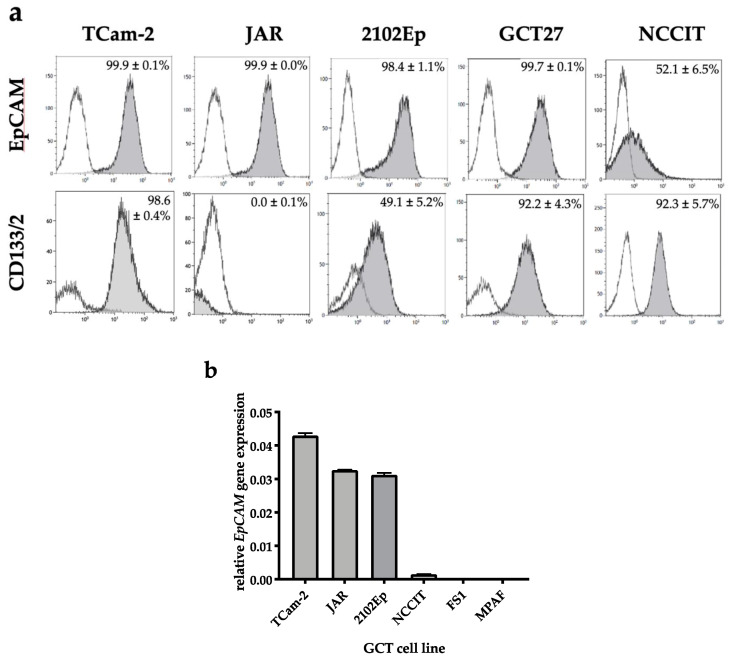
Expression of Epithelial Cell Adhesion Molecule (EpCAM) and CD133 mRNA and protein on the cell surface is detectable in germ cell tumours (GCT) lines of different histologies. (**a**) EpCAM and CD133 proteins (grey peak of the histogram) on the tumor cell surface of five GCT cell lines were assessed by flow cytometry in comparison to the isotype control (transparent peak). The fraction of antigen-positive cells (mean ± SD) of 4–5 independent experiments is shown in percent in the upper right of each histogram. (**b**) Quantitative real-time PCR analysis of EpCAM mRNA expression in four GCT cell lines as well as in sertoli (FS1) and fibroblast (MPAF) control cells. Relative EpCAM gene expression levels were normalized against GAPDH and presented as 2^−Δct^ values. Samples were analyzed in triplicates.

Levels of EpCAM protein on the surface of different GCT cell lines were assessed by flow cytometry (Figure 1a). EpCAM positivity is close to 100% in GCT cell lines of different histology derived from seminoma (TCam-2), choriocarcinoma (JAR), and embryonal carcinoma (2102Ep and GCT27). In contrast, in the pluripotent EC line NCCIT, only 50% of tumor cells displays EpCAM.

Likewise, in *EpCAM* expression analysis (Figure 1b), high levels of *EpCAM* mRNA are found in TCam-2, JAR, and 2102Ep, while *EpCAM* mRNA expression is low in the EC cell line NCCIT and negligible in nonmalignant Sertoli cells (FS1) and fibroblasts (MPAF). CD133, which combined with EpCAM can be indicative for cancer stem cells, is expressed to high levels on the seminoma cell line TCam-2 and the EC lines GCT27 and NCCIT. CD133 is detected only on half of the cells in the nullipotent EC line 2102Ep and is absent on the CHC line JAR (Figure 1a).

### 2.2. Marked Cytotoxicity in the EC Line 2102Ep Mediated by the Bispecific EpCAM/CD3 Antibody in the Presence of Peripheral Blood Mononuclear Cells Persists Across a Broad Range of Antibody Dilutions

**Figure 2:**
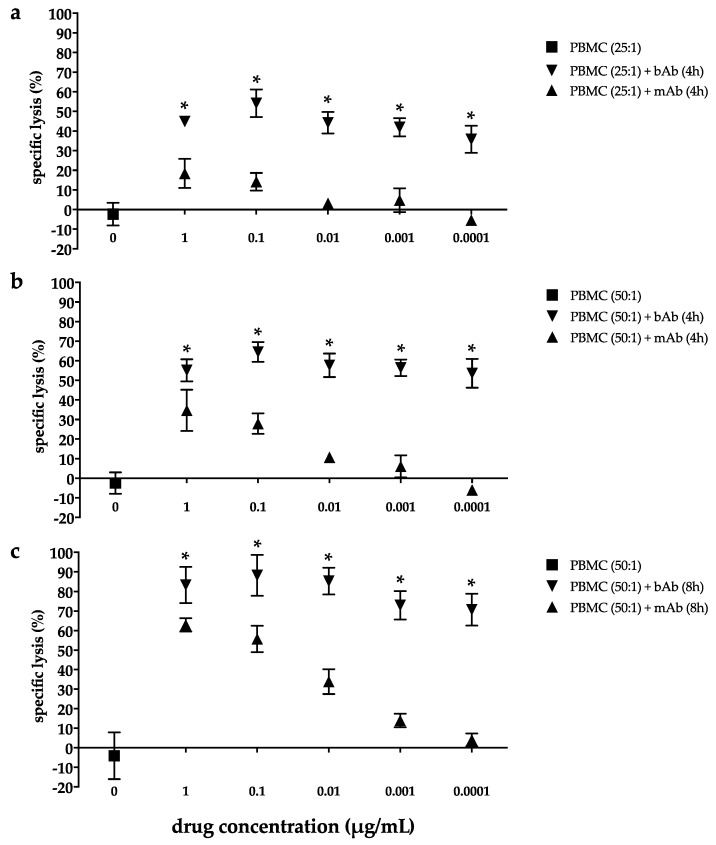
EpCAM/CD3-bispecific antibody mediates time-dependent strong cytotoxicity with stable activity at decreasing drug concentrations in the embryonal carcinoma cell line 2102Ep. 2102Ep cells were incubated for 4 h (**a**,**b**) or 8 h (**c**) with peripheral blood mononuclear cells (PBMC) at an effector:target cell ratio of 25:1 (**a**) or 50:1 (**b**,**c**) and stated concentrations of the monoclonal EpCAM-Ab Vu1D9 (mAB) or the bispecific trifunctional EpCAM/CD3-Ab Catumaxomab (bAb). Antibody-dependent cytotoxicity was assessed by europium release assay in triplicates and expressed in percentage of dead cells. Data are presented as mean ± SD of 2–3 independent experiments. Statistically significant difference between mAb- and bAb-mediated cell death is marked by an asterisk (* *p* < 0.001).

Cytotoxicity was assessed by europium release assay after treatment of the highly EpCAM-positive EC cell line 2102Ep for 4 h with different concentrations of peripheral blood mononuclear cells (PBMC; 25:1/50:1) including T, NK, and B cells as well as monocytes and either the bispecific trifunctional EpCAM antibody Catumaxomab (bAb) or the monoclonal EpCAM antibody Vu1D9 (mAb; Figure 2a,b).

PBMC alone had no cytotoxic effect on 2102Ep cells. In contrast, at an effector-to-target (E:T) ratio of 25:1, bAb-induced tumor cell lysis is 44.9 ± 2.5% at 1 µg/mL and 44.2 ± 5.4% at 0.01 µg/mL bAb. Even with further reduction of bAb concentration down to 0.0001 µg/mL, tumor cell lysis is still 35.8 ± 6.9% (Figure 2a). In the presence of the mAb, cytotoxicity is less pronounced across all drug concentrations (*p* < 0.001) and decreases with each dilution step. Thus, cell death is 18.4 ± 7.4% at 1 µg/mL and only 3.1 ± 2.1% at 0.01 µg/mL mAb.

Increasing the E:T ratio to 50:1 enhances both bAb- and mAb-mediated cellular kill (Figure 2b). Again, the EpCAM/CD3-bAb exhibits significantly more potent cytotoxicity than the mAb for all concentrations down to the lowest drug level (*p* < 0.001). Furthermore, cytolytic activity of the bAb persists at high levels across the entire drug concentration range, with 55.1% ± 5.7% at 1 µg/mL bAb and with 57.7 ± 6.0% and 53.6 ± 7.4% when treated with 0.01 µg/mL and 0.0001 µg/mL bAb, respectively. Upon incubation with the mAb in the presence of PBMC, only 34.7 ± 10.6% of 2102Ep cells die at 1 µg/mL and 10.7 ± 2.2% die at 0.01 µg/mL.

Prolongation of the incubation period further improves the cytotoxic effect of both the bAb and mAb (Figure 2c). Again, bAb-mediated cell death is marked and remains high despite decreasing drug concentrations. After incubation for 8 h in the presence of PBMC at an E:T ratio of 50:1, cell death is 83.3 ± 9.2% at 1 µg/mL bAb, 85.3 ± 6.8% at 0.01 µg/mL, and 70.7 ± 8.2% at 0.0001 µg/mL bAb. In contrast, cytotoxicity mediated by the mAb is significantly less pronounced across all drug concentrations (*p* < 0.001) and successively declines with each dilution step from 63.0 ± 3.4% at 1 µg/mL to 33.9 ± 6.4% at 0.01 µg/mL and only 4.0 ± 3.3% at 0.0001 µg/mL.

### 2.3. The EpCAM/CD3-Binding Bispecific Antibody Exerts Potent Cytotoxic Activity in GCT Cell Lines of Different Histologies

**Figure 3:**
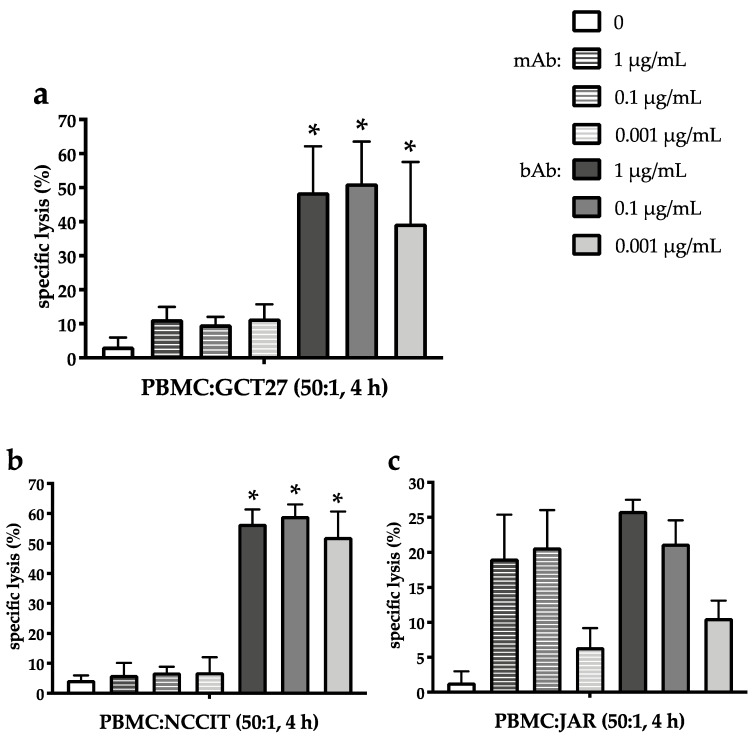
EpCAM/CD3-bispecific antibody exerts cytotoxic activity in different GCT cell lines independent of EpCAM expression and bAb concentration in the presence of PBMC. (**a**) GCT27, (**b**) NCCIT, and (**c**) JAR cells were incubated for 4 h with PBMC at an effector:target cell ratio of 50:1 and stated concentrations of the monoclonal EpCAM-Ab Vu1D9 (mAB) or the bispecific trifunctional EpCAM/CD3-Ab Catumaxomab (bAb). Antibody-dependent cytotoxicity was assessed by europium release assay in triplicates and expressed in percentage of dead cells. Data are presented as mean ± SD of two independent experiments. Statistically significant difference between mAb- and bAb-mediated cell death is marked by an asterisk (* *p* < 0.01).

Next, three additional histologically different GCT cell lines were incubated with EpCAM-recognizing bAb or mAb in the presence of PBMC at an E:T ratio of 50:1 (Figure 3a–c). Cytotoxicity was assessed by europium release. As in 2102Ep, the bAb exerts potent and dose-independent cytotoxicity in the EC cell line GCT27 expressing EpCAM to high levels (Figure 3a) with 48.7 ± 13.5%, 51.3 ± 12.3%, and 39.4 ± 18.0% of GCT27 cells killed at 1 µg/mL, 0.1 µg/mL, and 0.001 µg/mL bAb, respectively. In comparison, cytotoxicity mediated by the mAb is considerably lower in GCT27 for each drug level (*p* < 0.005) with only 11.4 ± 3.6% at 1 µg/mL, 9.9 ± 2.2% at 0.1 µg/mL, and 11.6 ± 4.1% at 0.001 µg/mL mAb (Figure 3a). 

Even in the pluripotent EC line NCCIT (Figure 3b) displaying EpCAM surface expression only on half of the tumor cells, the EpCAM/CD3-specific bAb still induces marked GCT lysis in the presence of PBMC, amounting to 56.5 ± 4.8%, 59.1 ± 3.9%, and 52.1 ± 8.5% at 1, 0.1, and 0.001 µg/mL, respectively. In contrast, cell death mediated by the EpCAM-binding mAB was constantly below 10%, i.e., 6.0 ± 4.0% at 1 µg/mL, 6.9 ± 1.9% at 0.1 µg/mL, and 7.0 ± 5.0% at 0.001 µg/mL mAb, and thus significantly inferior across all drug concentrations (*p* < 0.001).

Of note, in the CHC cell line JAR, bAb-induced cellular kill does not exceed mAb-mediated cytotoxicity (Figure 3c). In spite of EpCAM expression close to 100% of tumor cells, JAR lysis triggered by the EpCAM/CD3-bAb reaches only 25.9 ± 1.6% at 1 µg/mL and 21.2 ± 3.3% at 0.1 µg/mL bAb. Also compared to EC lines, bAb-mediated cytotoxicity is not preserved down to the lowest bAb-concentrations as only 10.6 ± 2.5% of JAR cells are killed at 0.001 µg/mL bAb. Cellular kill mediated by the mAb reaches 19.1% ± 6.3% at 1 µg/mL and 20.7% ± 5.3% at 0.1 µg/mL mAb. Again, only 6.4 ± 2.7% of JAR cells are eradicated at the lowest mAb concentration of 0.001 µg/mL mAb. Thus, at all tested drug concentrations, no statistically significant difference in the efficacy of antibody-mediated tumor cell death is observed between mAb and bAb (1 µg/mL: *p* = 0.23; 0.1 µg/mL: *p* = 0.18; 0.001 µg/mL: *p* = 0.15).

### 2.4. NK Cells Contribute to Cytotoxic Efficacy of the EpCAM/CD3-bAb at Higher Drug Concentrations While T Cells Still Induce Apoptosis Even at Lower bAb Dosages

**Figure 4:**
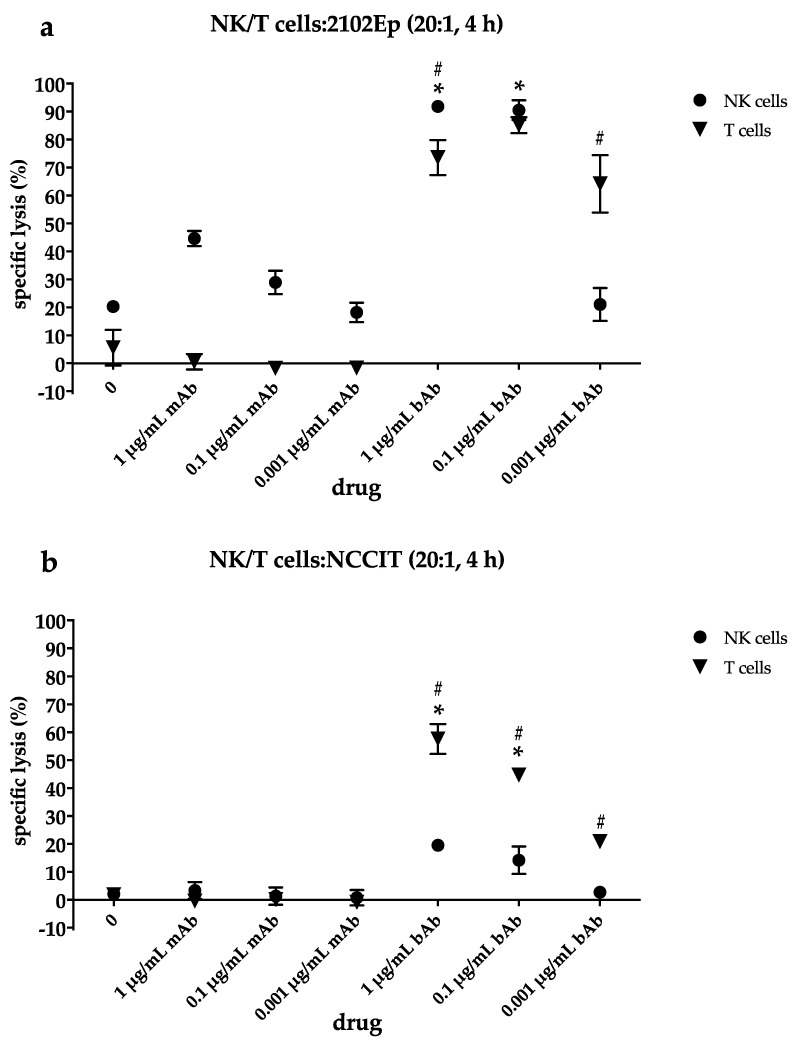
In the presence of either isolated natural killer (NK) and T cells, EpCAM expression exerts significant influence on the extent of bAb-mediated tumor cell lysis. (**a**) 2102Ep or (**b**) NCCIT cells were cultivated for 4 h with isolated NK or T cells at an effector:target cell ratio of 20:1 and stated concentrations of monoclonal EpCAM-Ab Vu1D9 (mAB) or the bispecific trifunctional EpCAM/CD3-Ab Catumaxomab (bAb). Antibody-dependent cytotoxicity was assessed by europium release assay in triplicates and expressed in percentage of dead cells. Data are presented as mean ± SD of two independent experiments. Statistically significant difference between mAb- and bAb-mediated NK cell-induced cellular lysis is marked by an asterisk (* *p* < 0.001). The hash (#) indicates a statistically significant difference between NK or T cell-induced cell death mediated by the bAb (*p* < 0.001).

To allocate antibody-mediated cytotoxicity to different effector cell subsets, NK and T cells were isolated from buffy coats and incubated separately at an E:T ratio of 20:1 either with the highly EpCAM-positive EC line 2102Ep or the EC line NCCIT expressing EpCAM only on 50% of tumor cells. Cytotoxicity was assessed by europium release assay after 4 h of incubation with the bispecific and monospecific antibodies (Figure 4a,b).

In the absence of a specific binding site for T cells, EpCAM-mAb facilitates only NK but no T-cell dependent cytotoxicity. In the EpCAM-“high” EC line 2102EP, mAb-mediated cell death is substantial at the highest mAb concentration in the presence of NK cells but declines from 44.6 ± 2.7% at 1 µg/mL mAb to 28.9 ± 4.1% at 0.1 µg/mL and 18.2 ± 3.5% at 0.001 µg/mL mAb (Figure 4a). The bispecific EpCAM/CD3-binding antibody engages isolated NK cells to an even more powerful cellular kill at the first two dose levels (*p* < 0.001). Thus, cell death in 2102Ep reaches 91.8 ± 0.7% at 1 µg/mL bAb and 90.5 ± 3.5% at 0.1 µg/mL bAb. Further reduction of the bAb concentration results in a drop of NK cell-mediated cell death down to 21.1 ± 5.9% at 0.001 µg/mL bAb, which is no longer statistically different to NK cell-mediated cell death in the presence of mAb at this concentration (*p* = 0.43).

In contrast, following incubation with isolated T cells (Figure 4a), induction of cell death persists at high levels despite decreasing bAb concentrations. In the presence of EpCAM/CD3-bAb, cytotoxicity in 2102Ep cells amounts to 73.6% ± 6.3% at 1 µg/mL bAb and 85.1 ± 2.9% at 0.1 µg/mL bAb and is still 64.1 ± 10.3% at 0.001 µg/mL bAb, resulting in significantly better T cell- compared to NK cell-mediated cellular killing at the lowest drug level (*p* < 0.001).

In NCCIT with only 50% of EpCAM-positive cells, the EpCAM-binding mAb fails to mediate relevant NK cell-mediated cytotoxicity. Even at the highest concentration of 1 µg/mL mAb, only 3.4 ± 3.0% of tumor cells are killed after incubation with NK cells (Figure 4b). In contrast, NK cell-mediated cell death induced by bAb is significantly more prominent especially at higher drug levels, with 19.6 ± 1.3% at 1 µg/mL bAb and 14.2 ± 5.0% at 0.1 µg/mL (*p* < 0.001 each). At 0.001 µg/mL bAb, cellular lysis is negligible with 2.7 ± 0.8% without statistical difference to the mAb at the same drug concentration (*p* = 0.9). In the EpCAM-“low” EC line NCCIT, T cell-dependent cytotoxicity in the presence of bAb significantly exceeds NK cell-mediated cell death (Figure 4b) at each drug level (*p* < 0.001). Cellular kill declines in a dose-dependent manner from 57.6 ± 5.3% at 1 µg/mL bAb to 44.7 ± 2.3% at 0.01 µg/mL and 20.8 ± 1.4% at 0.001 µg/mL bAb.

### 2.5. Phenotypic Characterization of Immunomodulatory Surface Molecules in GCT Cell Lines

**Figure 5:**
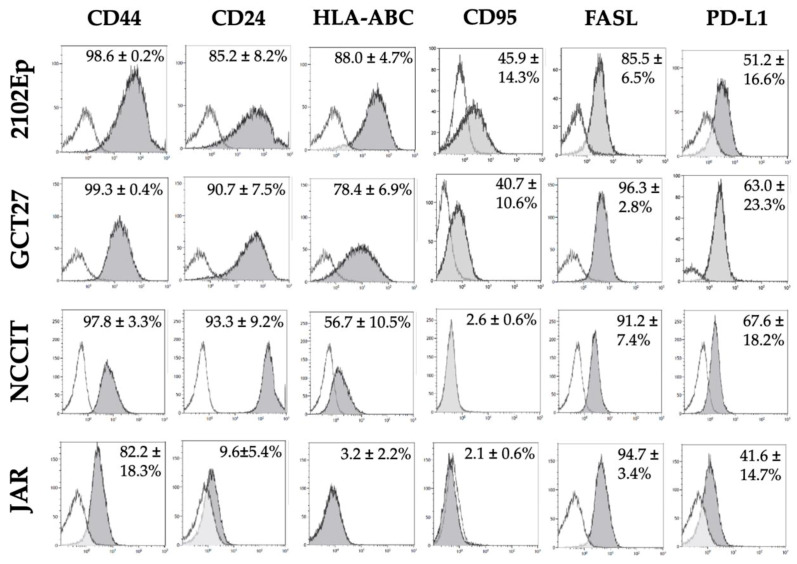
JAR lacks expression of antigens modulating the immune response. Expressions of different immune response-modulating proteins (grey peak of the histogram) on the tumor cell surface of four GCT cell lines were assessed by flow cytometry in comparison to the isotype control (transparent peak). The fraction of antigen-positive cells (mean ± SD) of at least three independent experiments is shown in percent in the upper right of each histogram (*n* = 3–7).

Expression of CD44 mediating T cell migration and adhesion is high in all GCT lines analyzed, while the costimulatory molecule CD24 is expressed to high levels on EC but is absent in the CHC line JAR (Figure 5). MHC-I expression is high on the EC lines 2102Ep and GCT27, intermediate in NCCIT, and absent in the CHC line JAR. With regard to T cell downmodulating ligands, all GCT lines are found to express FasL on their surface and PD-L1 in about 50% of tumor cells. Regarding susceptibility to extrinsic apoptosis pathways, it is of note that the Fas receptor is detectable on half of the tumor cells in the EpCAM-“high” EC lines 2102Ep and GCT27 and negligible in the pluripotent EC line NCCIT as well as the CHC line JAR.

## 3. Discussion

In GCT, diverse immune cell infiltration of the tumor microenvironment reflects the heterogeneous histology of this group of neoplasms, ranging from virtual absence of immune cells in CHC to intermediate T cell accumulation in EC and YST and to dense inflammatory immune cell infiltration in gonadal dysgerminoma and seminoma. Thus, immunotherapeutic agents such as bispecific antibodies that specifically target malignant cells and redirect T cells to the tumor microenvironment constitute a promising approach to harness the immune system for antineoplastic protection in GCT.

Here, we extend our previous findings of *EpCAM* mRNA and EpCAM protein levels in GCT of different histologies [17] and demonstrate profound in vitro vulnerability of various EpCAM-positive GCT cell lines to the cytotoxic effects of an EpCAM/CD3-bispecific antibody in the presence of immune cells. Thus, in three EpCAM-positive EC cell lines, more than half of the cells are killed within the first 4 h of incubation with EpCAM/CD3-bAb in the presence of PBMC. After prolonged exposure, cytotoxicity reaches more than 80% in the highly EpCAM-positive EC cell line 2102Ep. Amongst bispecific antibodies, the EpCAM-binding bAb Catumaxomab represents the prototype of a bispecific T cell-redirecting antibody and is the first bAb to be licensed for clinical use by the European Medicines Agency (EMA), even if application of this rodent-derived antibody was ultimately confined to intraperitoneal use due to systemic immunogenicity [22]. Catumaxomab has documented efficacy in a variety of EpCAM-overexpressing epithelial cancer subtypes like ovarian, breast, and gastric carcinoma [23,24]. Of note, high EpCAM expression has been identified as an independent parameter indicative of chemoresistance in an immunohistochemical analysis of 168 primary ovarian cancer tissues [25]. Conversely, EpCAM knockdown by siRNA sensitizes eosophageal adenocarcinoma tumor spheres to cisplatin-based chemotherapy in vitro [26]. Here, we document marked cytotoxicity in less differentiated, partially pluripotent EpCAM-positive GCT cell lines, demonstrating that not only epithelial but also non-epithelial GCT subsets represent suitable targets for future clinical exploration of EpCAM-directed immunotherapy.

It is noteworthy that, in GCT of different histologies, cytotoxicity facilitated by the EpCAM/CD3-binding bAb Catumaxomab is stable over a broad range of antibody concentrations from 1 µg/mL down to 0.0001 µg/mL, the latter corresponding to plasma levels achieved after intravenous administration of the lowest dose in a phase I dose-escalation study [27]. In vitro, upon recruitment of T cells via the anti-CD3-arm and crosslinking of accessory immune cells by the Fc-region, production of an array of immunomodulating cytokines such as IL-1β, IL-2, IL-6, IL-12, and DC-CK1 has previously been documented [28]. In effector cells harvested from ascites of patients suffering from EpCAM-positive cancers, the bAb has further been shown to induce effector cytokines such as interferon-γ and upregulation of surface-expressed CD107a indicative of cytotoxic granule release in both CD4+ and CD8+ T cells [29]. Furthermore, engagement of NK cells via its Fc-receptor results in expression of TRAIL, a facilitator of the granule-independent mode of cellular kill. Also, the C-type lectin receptor CD69 is upregulated, known to promote NK cell activation and expansion [29]. Taken together, antineoplastic efficacy of the bispecific EpCAM/CD3-binding antibody is based on the interplay of T cells and activated accessory cells. This is in keeping with our observation that, in the presence of PBMC, a cellular mix of lymphocytes, NK cells, and monocytes, cytotoxicity triggered by the EpCAM/CD3-binding bAb is substantial and largely independent of the extent of target antigen expression and antibody concentration. In contrast, in the presence of isolated T or NK cells alone, the density of EpCAM surface expression exerts an influence on the extent of bAb-mediated tumor cell lysis. Thus, in the pluripotent intermediate EpCAM-positive EC cell line NCCIT, cytotoxicity is inferior in the presence of either immune cell subset compared to the nullipotent and EpCAM-“high” EC line 2102Ep. Of note, in the highly EpCAM-positive EC cell line 2102Ep, T cell-mediated cell death is pronounced and persists at high levels over a four-log range of antibody dilution. 

In contrast, in the CHC line JAR, the bAb fails to trigger tumor cell lysis by isolated T cells alone whilst equivalent cytotoxic efficacy of the bispecific and monoclonal antibody indicates predominantly NK cell-mediated tumor cell destruction. As a consequence, the overall sensitivity of the CHC cell line JAR to antibody-mediated lysis is limited. MHC subclasses can function as killer cell inhibitory receptors, and their absence as a phenotypic characteristic in CHC might contribute to promoting NK cell activity whilst impeding T cell responses. In addition, lack of MHC-I as well as costimulatory molecules is one of the key mechanisms by which tumor cells escape antigen-specific immune surveillance. In contrast to EC lines, JAR lacks CD24 expression, a key factor of T cell homeostasis and proliferation [30]. Choriocarcinoma cells have also been shown to downregulate lymphocyte-production of IL-2 [31], a cytokine involved in the expansion of T and NK cell responses. With regard to target cell sensitivity, it is of note that JAR fails to express CD95 as the binding site for Fas ligand, thereby circumventing induction of extrinsic apoptosis via Fas/FasL interaction as previously described in two other CHC cell lines [32]. Downstream, the apoptotic inhibitor survivin and other antiapoptotic proteins of the XIAP family are strongly expressed in CHC, potentially counteracting proapoptotic signals delivered via bAb-crosslinked T cells [33]. Altogether, this may contribute to the low rate of cellular destruction despite high-level EpCAM expression in JAR.

In many cancers, PD-1/PD-L1 expression on neoplastic and other cellular components of the tumor microenvironment has been recognized as key to tumor cell-mediated suppression of the antineoplastic immune response. Immunohistochemical analysis of testicular GCT revealed variable expression of PD-L1 and CTLA-4, with PD-L1 expression in GCT being significantly higher compared to normal testicular tissue [34]. In addition, CTLA-4 positivity is frequent in GCT-infiltrating immune cells [35]. However, in GCT, clinical studies of single immune checkpoint inhibition with PD-1/PD-L1 or CTLA-4 blockade has so far been of limited benefit with efficacy confined to GCT of an inflamed phenotype [14,15], such that more recently, combinations of checkpoint inhibitors, i.e., Nivolumab (anti-PD-1) and Ipilimumab (anti-CTLA-4), have come into focus.

Concerning bAb-treatment, this is of interest as, similar to attenuation of MHC-dependent cytotoxic T cell responses, T cells redirected to EpCAM-positive target cells may become subdued by various mechanisms of immune inhibition such as PD-1/PD-L1 and CTLA-4/CD80/86 [36]. Indeed, treatment with EpCAM/CD3-bAb in a murine melanoma model resulted in upregulation of the immune checkpoint molecule CTLA-4 on recruited T cells in vivo and CTLA-4 blockade enhanced the humoral antimelanoma response with moderate survival benefit [37]. Combination of checkpoint inhibition and redirected T cell-mediated tumor cell lysis by bAbs is therefore deemed promising. Currently, a phase I study evaluates the combination of the PD-1 inhibitor Pembrolizumab and the PMSA/CD3-bAb AMG160 in metastatic castration-resistant prostate cancer (NCT03792841). In view of the pertinent influence of active WNT signaling on the immunological tumor microenvironment [20,21], combination of immunotherapeutic strategies such as bAb treatment with inhibitors of the WNT pathway are forthcoming and may in the future prove to be of particular relevance in GCT considering the pertinent role of the WNT cascade in GCT.

GCT, despite their characteristically low mutational burden and lack of neoantigens, express proteins characteristic of their embryonal origin such as Alpha-fetoprotein (AFP) with documented MHC-I-restricted, AFP-directed antineoplastic T cell responses in other AFP-positive cancers [38,39]. The notion that bispecific antibodies harbor the potential to induce long-term immunological protection in addition to acute tumor cell lysis has been exemplified in a murine model of EpCAM-positive melanoma. In this model, after a first melanoma challenge in the presence of an EpCAM/CD3-bAb, endogenous EpCAM-specific antibodies were induced in a T and accessory cell-dependent manner and mice survived subsequent tumor challenges without repeated antibody treatment while use of bispecific F(ab’2) fragments lacking the Fc portion failed to induce memory formation [40]. Translating this observation to the clinical setting, primary treatment of EpCAM-positive GCT with EpCAM/CD3-binding trifunctional bAb not only might reduce initial tumor burden but also may contribute to preventing subsequent GCT relapse.

Broad activation of cellular immunological effectors however carries the risk of nonspecific off-target effects. Thus, following treatment with the bAb Catumaxomab, it is the nonspecific binding of the Fc-domain to FcγR-positive Kupffer cells that was found to be the cause for the observed antibody-mediated hepatoxicity [41]. Bispecific T cell engagers such as Solitomab that consist solely of single-chain variable fragments for binding of EpCAM and CD3 circumvent accessory cell recruitment. In primary ovarian cancer cell lines resistant to cisplatin as well as to adoptively transferred immune effector cells, Solitomab was capable of inducing profound T cell cytotoxicity in vitro and in an ex vivo model of malignant ascites [42]. Clinically, Solitomab has demonstrated efficacy in EpCAM-positive colon cancer with gastrointestinal toxicity as a significant on-target side effect [43]. An intriguing approach to limit on- and off-target toxicity is the combination of redirecting bAb with adoptively transferred T cells genetically modified to express a marker as an alternative target for T cell binding. In case of excessive toxicity, T cells can be eliminated by administration of additional antibodies directed against the marker antigen expressed solely on the adoptively transferred T cells [44].

Likewise, in T cells retrovirally modified to express target antigen-specific chimeric T-cell receptors (CAR), incorporation of suicide genes such as inducible caspase-9 in vector constructs allows for optional induction of T cell apoptosis to this end [45]. Indeed, third-generation EpCAM-redirected CAR-T cells have been documented to specifically recognize and to kill different EpCAM-positive ovarian cancer cell lines [46]. In GCT, CD30-redirected CAR-T cells have been shown to exert profound antitumor activity in CD30-positive EC cells both in vitro and in an EC xenograft [47]. Of note, in this model, bystander efficacy was documented as CD30-redirected CAR-T cells also eliminated CD30-negative EC cells via Fas/FasL interaction [47]. We have previously shown that the CD30-directed antibody-toxin drug conjugate Brentuximab vedotin exhibits bystander cytotoxic activity following release of the uncoupled toxin after internalization of the antibody conjugate by successfully CD30-targeted GCT cells [48]. As GCT represent typical neoplasms of mixed histology and variable target antigen expression within a single tumor, such bystander activity is critical for therapeutic success.

Currently, there is a surge of development of bAb and CAR-T cells with over two hundred constructs in early clinical testing. Among these are two EpCAM-recognizing bispecific antibodies and two CAR-T cell constructs [22]. In view of future immunotherapy approaches particularly in cisplatin-resistant GCT, this is noteworthy as we have demonstrated here that an EpCAM/CD3-binding bAb exerts profound in vitro cytotoxicity in GCT of different histologies.

## 4. Materials and Methods 

### 4.1. Cell Culture

JAR (HTB-144), GCT27, and NCCIT were purchased from American Type Culture Collection (Manassas, VA, USA). TCam-2 and 2102EP cells were kindly provided by L.Looijenga (Princess Máxima Center for Pediatric Oncology/NL). Cell lines were cultivated as described previously [17,48].

### 4.2. Cytofluorimetric Analysis

Monoclonal antihuman-EpCAM (clone: 9C4; manufacturer: Biolegend, fluorescent protein: APC), -CD133 (293C3, Milteny Biotech, PE), -CD44 (IM7, eBioscience, PE), -CD24 (eBioSN3, eBioscience, PE), -HLA-ABC (W6/32, eBioscience, PE), -CD95 (DX2, Thermofisher, APC), -FasL (NOK-1, Biolegend, PE), and -PD-L1 (MIH1, Thermofisher, PE) as well as mouse-IgG1 antibodies as isotype controls (P3.6.2.8.1, eBioscience, APC; MOPC-173, Becton Dickson, PE) were used for flow cytometry. Collected cells were first washed with PBS + 2% FCS (FACS buffer), stained with the fluorochrome-conjugated monoclonal antibodies, and incubated for 15 min at room temperature. Upon washing to remove unbound reagents, cells were resuspended in FACS buffer, acquired on a Navios^TM^ flow cytometer (Beckman Coulter Inc., Brea, CA, USA), and analyzed with Cytometry List Mode Data Acquisition and Analysis Software (Beckman Coulter Inc.).

### 4.3. Quantitative Real-Time RT-PCR

Quantitative real-time RT-PCT (qRT-PCR) was performed as described previously [49]. The following primers were used to detect *EpCAM* expression: forward 5′-GCAGCTCAGGAAGAATGTG-3′, reverse 5′-CAGCCAGCTTTGAGCAAATGAC-3′. *GAPDH* was used as a housekeeper and for data normalization with the following *GAPDH* primers being employed: forward 5′-TGCCAAATATGATGACATCAAGAA-3′, reverse 5′-GGAGTGGGTGTCGCTGTTG-3′. A melting point analysis was performed to confirm primer specificity. PCR was performed at 94 °C/30 s and 60 °C/60 s for 40 cycles using the ViiA 7 Real-Time PCR System (Applied Biosystems, Foster City, CA, USA).

### 4.4. Isolation of Natural Killer and T Cells 

Blood samples from healthy donors were collected by the local institute of Experimental Haematology and Transfusion medicine (IHT) at the University Hospital of Bonn. Peripheral blood mononuclear cells (PBMCs) were separated by Ficoll–Hypaque density gradient centrifugation (specific gravity, 1.077 g/mL; Lympholyte^TM^, Cedarlane, Burlington, Canada). NK cells were negatively selected from PBMCs using the untouched NK cell isolation kit (human) onto LD columns (Miltenyi Biotec, Bergisch Gladbach, Germany), according to the manufacturer’s instructions. Purity of enriched NK cells was assessed by flow cytometry. For isolation of T cells, 10 mL of Buffy coat was incubated with 500 µL RosetteSep^TM^ Human T Cell Enrichment Cocktail (Stemcell Technologies, Vancouver, Canada) and proceeded according to the manufacturer’s instructions. 

### 4.5. Europium Release Assay

All chemicals for the Europium labeling buffer were of analytical grade provided by Sigma-Aldrich (Germany). Labeling buffer contained 50 mM 4-(2-hydroxyethyl)-1-piperazineethanesulfonic acid (HEPES), 93 mM NaCl, 5 mM KCl, 2 mM MgCl_2_, and 10 mM diethylenetriaminepentaacetic acid (DTPA, dissolved in NaOH) in 1 liter of distilled water and was adjusted to pH 7.4. Upon sterile filtration, 100 mM Europium(III)chloride dissolved in distillated water was added to the labelling buffer. Tumor cells (5 × 10^6^) from the GCT cell lines 2102Ep, GCT27, NCCIT, or JAR were incubated 10 min. on ice with Europium-labelling buffer. Then, target cells were permeabilized by electroporation using the Lonza Nucleofector^TM^ II with SCN Basic Neuro Program 8 (Lonza, Basel, Switzerland) and washed four times with RPMI 1640 20% FCS. 

The cytotoxicity assay is based on the Europium-DPTA-chelate (Eu-DPTA) release from the cytoplasm of lysed cells, which is quantified in the supernatant with a time-resolved fluorometer. Target cells labeled with Europium were adjusted to a concentration of 5 × 10^3^ cells/100 µL medium and dispensed into wells of 96-well roundbottomed microtiter plates. An equal volume of effector cells was added to each well. Suspensions of effector cells were adjusted to give effector/target (E/T) ratios ranging of 50:1 and 25:1 for PBMC and 20:1 for isolated NK and T cells, respectively. Monoclonal antihuman EpCAM-antibody (Vu1D9; Thermo Fisher Scientific, Waltham, MA, USA) or trifunctional EpCAM/CD3-antibody Catumaxomab (Fresenius, Bad Homburg, Germany) were added in concentrations ranging from to 0.0001 to 1 µg/mL. Controls consisted of PBMC and media without tumor cells. All assays were performed in triplicate. The microplates were centrifuged briefly to bring effectors and targets in contact with each other and then incubated for 4 h or 8 h at 37 °C in a humidified atmosphere of 5% CO_2_ in air.

After incubation, 20 μL aliquots of the supernatants were transferred to wells of a flat-bottom 96-well microplate (FluoroNunc^TM^, Sigma-Aldrich, St. Louis, MO, USA), and 200 μL aliquot of enhancement solution (DELFIA enhancer solution; PerkinElmer, Waltham, MA, USA) was added to each well. After mixing for 10 min., fluorescence was measured in a time-resolved fluorometer (VICTOR, PerkinElmer). The percentage of specific cytotoxicity was expressed as specific release in % ((experimental release−spontaneous release)/(maximum release−spontaneous release) × 100). Spontaneous release was determined by incubating the targets with 100 μL of complete medium instead of effector cells, and maximum release was determined by incubating the cells with 100 μL of 0.1% Triton-X. 

### 4.6. Statistical Analysis

Calculations of mean values, standard deviation, and *p*-values were performed using Prism 8 (GraphPad, San Diego, CA, USA). For determination of statistical significance between the cytotoxic efficacy of the bispecific compared to the monoclonal antibody, two-tailed Student´s *t*-test and multiple *T*-tests with adjusted *p*-values were used. *p*-values less than 0.05 were considered statistically significant.

## 5. Conclusions

Although prognosis in GCT patients with cisplatin as the mainstay of treatment is excellent, there remains a subgroup of patients for whom novel therapeutic approaches are required. We demonstrate that, in GCT, the EpCAM-directed prototypic bispecific antibody Catumaxomab facilitates recruitment and activation of accessory cells in addition to redirected T cells and promotes highly efficacious antineoplastic toxicity. We also delineate differences in GCT phenotype potentially favoring immune escape that are worth further investigation. Combined effector cell redirection and blockade of inhibitory mechanisms with checkpoint inhibitors may serve to elevate attenuation of redirected antineoplastic immune responses in the future. Based on our in vitro findings in GCT and early clinical experience of EpCAM-redirected immunotherapy in other epithelial EpCAM-positive cancer entities, such therapeutics may also prove beneficial for the treatment of cisplatin-resistant GCT and warrant clinical exploration.
